# Sequence variants in *COL4A1* and *COL4A2* genes in Ecuadorian families with keratoconus

**Published:** 2011-03-30

**Authors:** Justyna A. Karolak, Karolina Kulinska, Dorota M. Nowak, Jose A. Pitarque, Andrea Molinari, Malgorzata Rydzanicz, Bassem A. Bejjani, Marzena Gajecka

**Affiliations:** 1 Institute of Human Genetics, Polish Academy of Sciences, Poznan, Poland; 2 Basic Medical Sciences Program, WWAMI (Washington, Wyoming, Alaska, Montana, and Idaho), Washington State University, Spokane, WA; 3 Department of Ophthalmology, Hospital Metropolitano, Quito, Ecuador; 4 Signature Genomics, Spokane, WA

## Abstract

**Purpose:**

Keratoconus (KTCN) is a non-inflammatory, usually bilateral disorder of the eye which results in the conical shape and the progressive thinning of the cornea. Several studies have suggested that genetic factors play a role in the etiology of the disease. Several loci were previously described as possible candidate regions for familial KTCN; however, no causative mutations in any genes have been identified for any of these loci. The purpose of this study was to evaluate role of the collagen genes collagen type IV, alpha-1 (*COL4A1*) and collagen type IV, alpha-2 (*COL4A2*) in KTCN in Ecuadorian families.

**Methods:**

*COL4A1* and *COL4A2* in 15 Ecuadorian KTCN families were examined with polymerase chain reaction amplification, and direct sequencing of all exons, promoter and intron-exon junctions was performed.

**Results:**

Screening of *COL4A1* and *COL4A2* revealed numerous alterations in coding and non-coding regions of both genes. We detected three missense substitutions in *COL4A1*: c.19G>C (Val7Leu), c.1663A>C (Thr555Pro), and c.4002A>C (Gln1334His). Five non-synonymous variants were identified in *COL4A2*: c.574G>T (Val192Phe), c.1550G>A (Arg517Lys), c.2048G>C (Gly683Ala), c.2102A>G (Lys701Arg), and c.2152C>T (Pro718Ser). None of the identified sequence variants completely segregated with the affected phenotype. The Gln1334His variant was possibly damaging to protein function and structure.

**Conclusions:**

This is the first mutation screening of *COL4A1* and *COL4A2* genes in families with KTCN and linkage to a locus close to these genes. Analysis of *COL4A1* and *COL4A2* revealed no mutations indicating that other genes are involved in KTCN causation in Ecuadorian families.

## Introduction

Keratoconus (KTCN, OMIM 148300) is a non-inflammatory, usually bilateral disorder of the eye, characterized by progressive thinning and protrusion of the central cornea which results in altered refractive powers and loss of visual acuity [1]. The prevalence of the disease is estimated to be 1 in 2,000 individuals, and is the most common ectatic disorder of the cornea [1]. KTCN afflicts males and females in all ethnic groups [1]. Signs and symptoms depend on the stage of disease, with the first signs usually appearing in the third decade of life [1,2]. The cause of KTCN is still unknown; both genetic and environmental factors seem to play a role in its etiology. Although most cases of KTCN are isolated, an association with many syndromes, such as Down syndrome [3], Ehlers-Danlos syndrome [4], and Leber congenital amaurosis [5] has been described. Furthermore, extensive studies have shown an association between KTCN and constant eye rubbing [6], contact lens wear [7], or atopy [8]. Usually, KTCN is a sporadic disorder, but positive family history has been observed in 6%–8% of cases [1]. An autosomal dominant inheritance pattern with reduced penetrance has been suggested in 90% of patients with familial KTCN [9,10].

Genomewide linkage analyses have indicated several loci involved in the etiology of familial KTCN at 16q22.3-q23.1 (KTCN2; OMIM 608932), 3p14-q13 (KTCN3; OMIM 608586), 2p24 (KTCN4; OMIM 609271), 1p36.23–36.21, 5q14.3-q21.1, 5q21.2, 5q32-q33, 8q13.1-q21.11, 9q34, 14q11.2, 14q24.3, 15q2.32, 15q22.33-q24.2, 17p13, and 20q12 [10-20]. However, no mutations in any genes at any of these loci have been associated with KTCN.

We have demonstrated an evidence of linkage to a novel locus at 13q32 [21]. Collagen type IV, alpha-1 (*COL4A1*; OMIM 120130) and collagen type IV, alpha-2 (*COL4A2*; OMIM 120090) are mapped in close proximity to that locus. The *COL4A1* and *COL4A2* genes are organized in a head-to-head conformation [22]. These gene pairs share a common promoter, and each gene is transcribed in opposite directions [23]. The *COL4A1* gene is placed on the minus strand and consists of 52 exons, while the *COL4A2* gene is on the opposite strand and consists of 48 exons. They encode two of six collagen type IV chains – α1 and α2 (1,669 and 1,712 amino acids, respectively) – forming a heterotrimeric protein molecule of collagen type IV (α1α1α2), which is found in the structure of the basement membrane (BM) [22,23]. Each chain contains three domains: an NH_2_-terminal 7S domain, a major collagenous domain with Gly-X-Y repeats (the X position is frequently occupied by proline, whereas the Y position is often occupied by 4-hydroxyproline) and a non-collagenous domain (NC1) at the COOH-terminus. Repetitions of the Gly-X-Y motif determine the formation of the triple-helical structure of collagen [22].

Collagens are the major protein components of the human cornea, and several types of collagen, including collagen type IV, have been identified [24]. Biochemical studies have revealed thinning of corneas from patients with KTCN, which may occur as a result of a reduced amount of total collagen proteins [25] and changes in collagen fibers orientation [26]. Moreover, a cornea affected by KTCN contains defects in BM and alterations in the BM composition [27]. The presence of collagen type IV in normal human cornea has remained unclear [28]. Results from expression arrays have shown an expression of *COL4A1* in transplant-quality human donor corneas [29] and a downregulation of *COL4A1* in keratoconus corneas [30]. Immunohistochemical studies have found collagen type IV α1/α2 chains in keratoconus corneas in large defect sites [28]. In light of these results, we recognize *COL4A1* and *COL4A2* as candidate genes for KTCN.

The purpose of this study was to screen *COL4A1* and *COL4A2* genes and determine whether sequence variants in these genes are involved in the causation of KTCN in Ecuadorian families.

## Methods

### Subjects

Twenty-three individuals from family KTCN-014, 25 affected individuals from other Ecuadorian families with KTCN, and 64 Ecuadorian control subjects were included in the study. The pedigrees of these families have been described elsewhere [21]. All individuals were examined in the Hospital Metropolitano in Quito, Ecuador, undergoing a complete ophthalmic evaluation as previously described [21]. The possible consequences of the study were explained and informed consent was obtained from all family members, according to the Declaration of Helsinki. Study protocol was approved by both the Institutional Review Board at Washington State University Spokane, Spokane, WA and Poznan University of Medical Sciences (Poland).

### Sequencing analyses

Oligonucleotide primers were designed to amplify all coding sequences and intron-exon junctions, promoter, and UTRs of both *COL4A1* and *COL4A2* (Table 1). PCR amplifications were performed using *Taq* DNA Polymerase (Fermentas Inc., Glen Burnie, MD). PCR products were purified with ExoSAP-IT® (USB Corporation, Cleveland, OH) or Montage® PCR Filter Units (Millipore, Jaffrey, NH) and sequenced using the BigDye® Terminator v3.1 Cycle Sequencing Kit (Applied Biosystems, Inc. [ABI], Foster City, CA). Sequencing was visualized on an ABI PRISM*®* 3100 Genetic Analyzer (ABI) and a 3730xl DNA Analyzer (ABI). The DNA sequences of study subjects were compared with the reference sequences of *COL4A1* and *COL4A2* (GRCh37/hg19, GenBank accession numbers for the mRNA NM_001845.4 and NM_001846.2, respectively) using Sequencher® 4.1.4. Software (Gene Codes Corporation, Ann Arbor, MI).

**Table 1:** Primer sequences and annealing temperature used to PCR amplifications of *COL4A1* and *COL4A2* fragments.

**Name**	**Forward**	**Reverse**	**Annealing Temperature (°C)**	**Amplicon Size (bp)**
COL4A1.1	CACCCTCCCCCTTTCTACTC	GCCCAGAGAATGCACCTG	59	837
COL4A1.2	TTGGGCTGAGTAACACTTGG	GCCTGGTTTGGCTTCATTTG	58	459
COL4A1.3–4	GGGCAACAGAATGAGACTCC	TGTGAGCTGGGAGAGGAGAT	66	477
COL4A1.5–6	TGCTCTGTCTGCTTTGTGTG	ACAAGCTGTGCTACTGGGTA	60	698
COL4A1.7–8	CCAACAAATGAAGGGTAGGG	TGTGCCAAGTGTCTGAACG	58	578
COL4A1.9–10	CCTTTGCTTTGCCGTCTCTA	TCATCATCCCTTTCCCACAG	60	691
COL4A1.11	GGAGATGGATTGGTATTGGT	GACTAAGGGATGGATGAAAG	58	451
COL4A1.12	GGGACAAAGCTATTGCCTGA	GACATTGATCCAAAGGTGGG	58	239
COL4A1.13	GCAGAGGCAAGGATGATTAG	GGGGCTCGTATTTTATGGAC	58	393
COL4A1.14–15	CCCTGCCCTGCTTACATT	GTCCCTACGAGCCTTTTCTG	60	505
COL4A1.16–17	TTAGTGGAGACGGGATTTCG	AACTGCCTGCTTGTGTATGC	60	725
COL4A1.18	GATGGGACAAGTATCTGGGC	CATCTCCTCTCCTTCCTCTC	60	459
COL4A1.19	GCTACCATTGCTGCTACTTCAC	AATAGAAAGCGTGGGGAGAG	62	447
COL4A1.20	GTCACAACAGGCTTCAGGAG	CCCAGGAGAGACATAAGGGT	60	486
COL4A1.21	CAGTGATGGTCTGGTTGGAT	ATGCCAGGAGTCTCAGAGGT	60	532
COL4A1.22	TGGGTGGTGTGTGGTGATTA	GAGAAGGGGCAAAACTCTGA	60	516
COL4A1.23	TTCCACCCATTAGCAGAGAG	GCCAACACACCAAAGCAA	60	304
COL4A1.24	GTCCGTCTTGGGCATTTTAG	ATTTGGGCTCTGTGGGTAAC	60	431
COL4A1.25	GTGCCCAAAGCCACACTATA	TGTTCAGTTCCCCCAAATGC	60	718
COL4A1.26	CCTGGGAGGGTAGATGAAGT	GAAAGGGAGGCACAAAAGG	62	488
COL4A1.27–28	AAGTGGAGAACACAGGCAGA	TCTTCCCAACCAAACCCTAC	56	636
COL4A1.29	AGGTGCTGGAAGAGACAGCA	GCTGAGGCTGAGAAACCATC	60	678
COL4A1.30	GCTTGAAAAGGGTTGAGCAG	GGCCTCTAAGATTTGCATCG	64	315
COL4A1.31	CAGAGCCCCTACCGAGTATA	CAGTGGGTGGGAGAAGAATC	61	483
COL4A1.32–33	CATTCAAGTTCCCAGTGTGG	GCCTTCTGCTTGATGTTCCT	60	653
COL4A1.34	CTCATTTACCTGGGGTTGGA	TATGGAGGACCCGATAACCC	60	411
COL4A1.35–36	TGTGCCTTTCCTGGGTTATC	AATGTCATCCATCCCTGAGC	64	594
COL4A1.37	GGGGGATTCACGTTCTTGTA	TCCCTGTGTGTTATGGCTCA	58	364
COL4A1.38–39	TGGCAGGTAGAAACCAGATG	TGAAGATGGGAGACAGGACA	61	641
COL4A1.40	GACCTCAGGAAAACCAGGTG	GTAGTTGCAGGGATGTGCAG	60	359
COL4A1.41	TGGTGGTTCTGAGCTGAAAG	CATGTGTCTTGCAGGCATTG	60	447
COL4A1.42	TAAAGAGAAGGAGGGATCGG	TCTTCACCAGAACCCACAAG	60	673
COL4A1.43	CCTGCCTCGATTTCTGTCTC	TAGTGGGGATGTGGGAGTGT	60	435
COL4A1.44	CCACAAGGCACCATTTGTTC	TACAAATTGGGCTGCCACAC	60	376
COL4A1.45	GGACCAAAAACAGTGCCCTA	GAGCCTTGGGAAGTTTCTGA	60	790
COL4A1.46	CCAGAATGCAGTGGGAAGTT	TTCCTGGGTTTTCTTCTGGA	60	590
COL4A1.47	ACAGCAAGAAACCAGGGAGA	GGCTGCCTTTCAACAACATC	60	591
COL4A1.48	TGAAGGAGGTAGGCTGCTGT	CGCAGTGTTTCACTCGCTAC	60	516
COL4A1.49	TGTTGTGAAAGACATTGCCC	GCCCAGCCAACTGACTTTTA	60	650
COL4A1.50	AAAACCAACGGGGAGGTACT	TAAGCAGCGAGATGCAGAGA	59	407
COL4A1.51	GGAAGCAGCCATTAGACGAT	AAATCGTCTCGGTCATCTGC	60	573
COL4A1.52.1	TACCAGGTTGAGGCCTGATG	ACCTCCTAGCACCCTTTGGT	65	530
COL4A1.52.2	GAAAACCAAAGGGTGCTAGG	CCGAATGTGCTTACGTGTGA	65	793
COL4A1.52.3	CCTGGCTTGAAAAACAGCTC	AATCACCCCCAGTCTGTGAC	60	429
COL4A2.1	TCGTGGGAAAGCTCAGATAC	AGACAAAGCGAGTTTAGCGC	60	1454
COL4A2.2	GCTTCTGGAAGGGCCAAT	GGGAAAGGGAGGAAGAGAGA	60	587
COL4A2.3	CCTCATCCTGCGCTAAACTC	ACACTTTCCTGGCCTCTACG	60	625
COL4A2.4	ATTTCAGGGGTGGGAGAGAC	CGGCCATCTAGGTTTGTGTG	60	467
COL4A2.5–6	TTCTTTCATCCCAACCCAGT	TCCCCACGTGTTTTATGTCA	59	663
COL4A2.7	AGACAGAAGAAACCCCGACA	TCTTGGGCGTCAACATACAG	60	515
COL4A2.8	TCAGAATAACCCCCATCAGC	AACAGATCAGCCCTATCAGGAC	60	568
COL4A2.9–10	AGGTCCTGATAGGGCTGATCT	TAACTGGCAGAGAGCTGGTG	59	551
COL4A2.11	GCATCAGAAACCTCCATGC	ACATTGGCCTCCCTACAACA	59	556
COL4A2.12	TCCAATCTCAGCTCCCACTC	TGTCCTCACCTCCACCTTCT	60	548
COL4A2.13	GGAAACAACCCCACAGAAAC	GGAGGACCCGGTTATGTTTT	59	524
COL4A2.14	GTAAACATCTGCCTGGAACG	CTATGGACAAGGGGATGAGA	58	469
COL4A2.15	TGTCACTGCCTGTCCTCAGA	CCCCAGTGCTAGATGTTCGT	61	513
COL4A2.16	ATTATTTCCCATCCCCACCT	GCAAAAATGAGAGCCAAGGT	59	473
COL4A2.17	CCCAGTGTCTTCAACAACCA	TGTCAGAGGCCGTGTATTTG	59	505
COL4A2.18	AGCACAGTCTCCTGGCATTC	CAGGCAACATGAAGGTCTCC	60	569
COL4A2.19	TTCGAGCTTTGGACTCACCT	CTGTGAAGGTGTCCAAAGCA	60	521
COL4A2.20	ACCCATCGGAGTTATTGACG	TACAGGGCTTCAGCTTCCAT	60	490
COL4A2.21	CCTGCATCTGTGGTTGTCTC	AAGTTCGCCTCCTCATCAAC	59	609
COL4A2.22	CCTCTGAATGTGGTCCCAGT	AAAGTCCGCCTTGGGGTAT	59	602
COL4A2.23–24	ATCGCAGAAAGTGCTCCTTG	ATGAGCAGCCTGTCCTATGC	60	545
COL4A2.25	TGGCACTAGGTTCCTGTTCA	ACAGGAGAGGCTGCATGTTT	59	553
COL4A2.26	AAACATGCAGCCTCTCCTGT	TTCTGACAAGAGGGGTTTGG	60	492
COL4A2.27	CCAGAATGGTAGCCGGTTT	GCAAGACCAGTTTGTGCTGA	60	318
COL4A2.28	TAAGCCTGGAGGTGCTGTTT	CCGAAACACCTGTCTCCTTT	59	499
COL4A2.29	GCGAAGGTTGTAGGTTCCAA	TGCCAAGACAAACAGTGAGC	60	708
COL4A2.30	GAATAGACAAGGGCAGGAAGG	CAGAGGATGAGCCGATGTCT	60	581
COL4A2.31	CACAGCCTCAACCTCCAGAT	CAGGCAGGAGCAGTTTGTCT	60	643
COL4A2.32	TGCTCCTCTGCCTTTGTCTT	TGTTGAGGCAGGGATAAAGC	60	656
COL4A2.33	TGGTCTCTCTCCAAGGCTTC	ACCGAGGTTACTCAGGCATC	59	442
COL4A2.34	ACAGCACGTAGGACAGCAAA	ACATCTGCATGGTGTCCAAG	59	470
COL4A2.35	GCTAAGCAAACCGCCTATGA	ACAGGACTTTCCACTGGGACT	60	416
COL4A2.36	GGGAGTCCACAATTCAGAGC	GACCCTTCGCTGTTTCTGAG	59	629
COL4A2.37	CCCATGCTTCTCTCCAATTC	ATGCCTCTCTCCATTCCTGA	60	446
COL4A2.38	CTGCTGCTGCTTTCTGTGTT	CCTGTGCTGCTATGTTGGTG	60	626
COL4A2.39	GTGCTGTCCCACACATGAAA	AGTCCATTCAACCCAGCAAC	61	510
COL4A2.40	ATGGGCCTCGATCCTCTTAT	AAACCAGCTCTTTCCTGCAC	60	484
COL4A2.41	CCCACCATGAGATGTTCCTT	ATGACACAGGAGGAGCCATC	60	427
COL4A2.42–43	AGTCATTCCATGCCACAGAC	TAAGCTCTCCATTCCCCAAG	60	666
COL4A2.44–45	CCCGTTAGTGTCTGGCTCAT	AGGTGTTCTGCTGGGCATAG	60	744
COL4A2.46	GAAACTGCCCTGCACTCCT	TAGATGGACCCTTCCGTCAG	60	664
COL4A2.47	CACTCCCTGGTGATCCAACT	CCAACTACCCTTGTGCAGTG	60	675
COL4A2.48.1	GGATGCCTCATGTCCGTATT	TACATGGGTGTGTGCGAAGT	60	689
COL4A2.48.2	CATCCAGCAGCAGCACTTAG	AGGTCTCCACTTCTGCCTGA	59	530
COL4A2.48.3	CCTGCTTTCTACGCCAATGT	CTGGTTGGGGTGTTTTCTGT	60	573

In the table, Amplicon Size represents length of the PCR product in base pairs (bp) and Annealing Temperature represents the annealing temperature of the primers used for PCR amplifications.

### Haplotype analysis

PEDSTATS [31] was used to verify the structure of KTCN-014 family and identify potential Mendelian inconsistencies in the inheritance of single nucleotide polymorphisms (SNPs) in *COL4A1* and *COL4A2*. For that region, to determine the full haplotypes inherited along with the substitutions occurring in affected individuals, a reconstruction of observed sequence variants was prepared using SimWalk2 [32,33]. Allele frequencies were set as equal. The location of genetic markers was determined on the basis of the Rutgers combined linkage-physical map of the human genome [34], either directly or by interpolation. Haplotype was generated with HaploPainter [35].

### Statistical analysis for Gln1334His substitution

The difference in distribution of Gln1334His substitution between affected and unaffected individuals in family KTCN-014 was analyzed by Fisher's Exact Test for Count Data. Similarly, 25 affected individuals from the remaining KTCN families versus 64 Ecuadorian control individuals were compared using Fisher's Exact Test. The difference between the examined groups was considered significant if the value of probability (p) did not exceed 0.05.

### Prediction of effect of amino acid substitutions on protein function

The potential impact of amino acid substitutions on the COL4A1 and COL4A2 proteins was examined using PolyPhen, SIFT, PMUT, PANTHER, and SNAP tools.

The PolyPhen tool predicts which missense substitution affects the structure and function of protein, and uses Position-Specific Independent Counts software to assign profile scores. These scores are the likelihood of the occurrence of a given amino acid at a specific position, compared to the likelihood of this amino acid occurring at any position (background frequency) [36].

The SIFT analytic tool, on the basis of gene sequences homology, evaluates conserved positions, and calculates a score for the amino acid change at a particular position. A score of <0.05 is considered as pathogenic and has a phenotypic effect on protein structure [37].

The PMUT calculates the pathological significance of non-synonymous amino acid substitution using neural networks (NN). NN output >0.5 is considered to be deleterious [38]. PANTHER estimates the likelihood of a particular amino acid’s change affecting protein function. On the basis of an alignment of evolutionarily related proteins, it generates the substitution Position-Specific Evolutionary Conservation (subPSEC). The subPSEC could achieve values from 0 (neutral) to about −10 (most likely to be deleterious). The value −3 is the cutoff point for functional significance, and corresponds to a P_deleterious_ of 0.5. If the substitution occurs at a position not appearing in the multiple sequence alignment, a subPSEC score cannot be calculated and change is not likely to be pathogenic [39,40].

The SNAP tool predicts the functional consequences of exchanging amino acids using evolutionary conservation and structure/function relationships. The SNAP output shows prediction neutral or non-neutral, and the expected accuracy [41].

## Results

Forty eight members of 15 Ecuadorian families and 64 Ecuadorian control subjects were included in the study. Twenty-three individuals from family KTCN-014, two affected individuals from each of the families KTCN-011, 015, 019, 020, 021, 024, 025, 030, 031, 034, and 035, and one patient from each of KTCN-05, 013, and 017 were examined.

### *COL4A1* and *COL4A2* sequence analyses

Screening of *COL4A1* (NM_001845.4) coding regions revealed 12 sequence variants, three of which were amino acid substitutions: c.19G>C (Val7Leu), c.1663A>C (Thr555Pro), and c.4002A>C (Gln1334His). We identified one novel synonymous change, c.3693G>A (Thr1231Thr), and eight previously reported sequence variants: c.432T>A (Ala144Ala), c.1257T>C (Pro419Pro), c.1815T>C (Pro605Pro), c.2130G>A (Pro710Pro), c.3183G>A (Gly1061Gly), c.3189A>T (Arg1063Arg), c.4470C>T (Ala1490Ala), and c.4800C>T (Ser1600Ser). In the 5′ untranslated region (5′ UTR), one novel sequence variant, c.84+124T>A, was identified. In the 3′ untranslated region (3′ UTR), two previously reported variants, c.*587C>A and c.*975A>C, were detected.

Sequencing analyses of *COL4A2* (NM_001846.2) coding regions revealed 13 previously reported sequence variants, including five non-synonymous substitutions: c.574G>T (Val192Phe), c.1550G>A (Arg517Lys), c.2048G>C (Gly683Ala), c.2102A>G (Lys701Arg), and c.2152C>T (Pro718Ser), and eight synonymous substitutions: c.297G>A (Thr99Thr), c.1008C>T (Pro336Pro), c.1095G>A (Pro365Pro), c.1179C>T (Ile393Ile), c.1488G>A (Pro496Pro), c.4089G>A (Ala1363Ala), c.4290T>C (Phe1430Phe), c.4515A>G (Pro1505Pro). In the 5′ UTR, five known nucleotide changes, c.-277A>C, c.-232C>G, c.-215C>T, c.-203T>C, and c.-133A>G, were identified. In the 3′ UTR, eight previously reported sequence variants, c.*76T>C, c.*101_*102del2, c.*417C>G, c.*541C>T, c.*557A>G, c.*650T>C, c.*663T>C, and c.*727G>C were detected.

Screening of exon/intron junctions in *COL4A1* and *COL4A2* revealed numerous sequence variants in the surrounding non-coding sequences, 71 and 86, respectively, including single nucleotide changes, insertions, and deletions. All screening results are summarized in Table 2.

**Table 2:** Sequence variants found in *COL4A1* and *COL4A2* genes.

					**Affected KTCN-014 (n=10)**		**Unaffected KTCN-014 (n=11)**		**Unknown KTCN-014 (n=2)**		**All KTCN-014 (n=23)**		**Other KTCN Families Affected (n=25)**		**All (n=48)**	
**Exon**	**dbSNP refID**	**Chromosome Position**	**Allele Change**	**Residue Change**	**no.**	**%**	**no.**	**%**	**no.**	**%**	**no.**	**%**	**no.**	**%**	**no.**	**%**
*COL4A1* (NM_001845.4)																
	-	110959464	c.-90G>T	-	5	50	7	63.6	0	0	12	52.2	15	60	27	56.3
1	rs9515185	110959356	c.19G>C	Val7Leu	8	80	6	54.5	1	50	15	65.2	18	72	33	68.8
	-	110959167	c.84+124T>A	-	10	100	11	100.0	2	100	23	100.0	25	100	48	100.0
	rs75270666	110895200	c.85–119C>T	-	0	0	2	18.2	1	50	3	13.0	5	20	8	16.7
	rs41275106	110895150	c.85–69T>C	-	1	10	3	27.3	0	0	4	17.4	4	16	8	16.7
	rs9521650	110866265	c.234+8C>T	-	2	20	1	9.1	0	0	3	13.0	5	20	8	16.7
	rs3737328	110866065	c.279+64G>A	-	5	50	5	45.5	1	50	11	47.8	10	40	21	43.8
7	rs532625	110864225	c.432T>A	Ala144Ala	7	70	8	72.7	2	100	17	73.9	14	56	31	64.6
	rs71805366	110863985, 110863989	c.468+5_468+9del5	-	3	30	2	18.2	0	0	5	21.7	4	16	9	18.8
	rs76574181	110862750	c.469–191C>T	-	3	30	4	36.4	1	50	8	34.8	4	16	12	25.0
	rs2166208	110862686	c.469–127C>T	-	3	30	4	36.4	1	50	8	34.8	4	16	12	25.0
	rs9521649	110862303	c.615+24C>T	-	3	30	6	54.5	1	50	10	43.5	16	64	26	54.2
	rs2166207	110862268	c.615+59T>G	-	7	70	9	81.8	2	100	18	78.3	21	84	39	81.3
	rs645114	110861785	c.616–11G>C	-	10	100	11	100.0	2	100	23	100.0	25	100	48	100.0
	rs7333204	110861671	c.651+68A>G	-	3	30	6	54.5	1	50	10	43.5	16	64	26	54.2
	rs7332120	110861670	c.651+69C>T	-	3	30	6	54.5	1	50	10	43.5	16	64	26	54.2
	rs10687642	110861652, 110861653	c.651+86_651+87ins2	-	3	30	6	54.5	1	50	10	43.5	16	64	26	54.2
	rs55833821	110861649	c.651+90C>G	-	3	30	6	54.5	1	50	10	43.5	16	64	26	54.2
	rs35638294	110861620, 110861621	c.651+118_651+119ins4	-	3	30	6	54.5	1	50	10	43.5	16	64	26	54.2
	rs7333008	110861560	c.651+179A>G	-	3	30	6	54.5	1	50	10	43.5	16	64	26	54.2
	rs598893	110859743	c.780+7G>A	-	7	70	8	72.7	2	100	17	73.9	17	68	34	70.8
	rs598819	110859690	c.780+60T>C	-	10	100	11	100.0	2	100	23	100.0	25	100	48	100.0
	rs9588116	110859069	c.808–7C>G	-	7	70	9	81.8	2	100	18	78.3	21	84	39	81.3
	rs67772891	110859326	c.781–88delT	-	10	100	11	100.0	2	100	23	100.0	25	100	48	100.0
	rs677877	110857895	c.859–10T>C	-	7	70	5	45.5	2	100	14	60.9	17	68	31	64.6
	rs482757	110857823	c.903+18G>A	-	7	70	5	45.5	2	100	14	60.9	17	68	31	64.6
	rs665713	110857502	c.957+198T>C	-	10	100	11	100.0	2	100	23	100.0	19	76	42	87.5
	rs648735	110856180	c.958–226T>C	-	7	70	8	72.7	2	100	17	73.9	17	68	34	70.8
	rs648705	110856153	c.958–199T>G	-	7	70	8	72.7	2	100	17	73.9	17	68	34	70.8
	rs7327728	110856094	c.958–140T>A	-	0	0	0	0.0	0	0	0	0.0	1	4	1	2.1
	rs648263	110856085	c.958–131T>C	-	7	70	8	72.7	2	100	17	73.9	17	68	34	70.8
	-	110855997	c.958–43delT	-	0	0	0	0.0	0	0	0	0.0	1	4	1	2.1
	-	110853032	c.1085–205A>T	-	10	100	11	100.0	2	100	23	100.0	22	88	45	93.8
	rs995223	110851036	c.1121–58A>G	-	3	30	6	54.5	1	50	10	43.5	17	68	27	56.3
	rs496916	110851014	c.1121–36C>G	-	10	100	7	63.6	1	50	18	78.3	8	32	26	54.2
21	rs995224	110850842	c.1257T>C	Pro419Pro	3	30	6	54.5	1	50	10	43.5	17	68	27	56.3
	rs683309	110850770	c.1285+44A>G	-	10	100	11	100.0	2	100	23	100.0	25	100	48	100.0
	rs9588112	110847566	c.1286–101G>A	-	3	30	4	36.4	1	50	8	34.8	4	16	12	25.0
	rs505050	110847227	c.1381+143C>A	-	10	100	10	90.9	1	50	21	91.3	21	84	42	87.5
	rs9521643	110847217	c.1381+153T>C	-	4	40	6	54.5	1	50	11	47.8	18	72	29	60.4
	rs2241966	110847190	c.1381+180A>G	-	4	40	6	54.5	1	50	11	47.8	18	72	29	60.4
	rs685884	110845314	c.1382–54C>T	-	9	90	11	100.0	2	100	22	95.7	21	84	43	89.6
	rs2241967	110844721	c.1466–90G>A	-	8	80	7	63.6	1	50	16	69.6	11	44	27	56.3
25	rs536174	110839550	c.1663A>C	Thr555Pro	10	100	11	100.0	2	100	23	100.0	25	100	48	100.0
	rs9521638	110839428	c.1728+57T>C	-	7	70	7	63.6	1	50	15	65.2	19	76	34	70.8
26	rs61749897	110838814	c.1815T>C	Pro605Pro	3	30	4	36.4	1	50	8	34.8	4	16	12	25.0
	rs2305080	110838703	c.1897+29A>G	-	7	70	7	63.6	1	50	15	65.2	19	76	34	70.8
	rs565470	110838646	c.1897+86T>C	-	9	90	10	90.9	2	100	21	91.3	25	100	46	95.8
	rs72654112	110835460	c.1991–16G>A	-	0	0	0	0.0	0	0	0	0.0	4	16	4	8.3
	rs7329411	110835195	c.2095+145G>T	-	7	70	7	63.6	1	50	15	65.2	19	76	34	70.8
29	rs16975492	110833702	c.2130G>A	Pro710Pro	7	70	7	63.6	1	50	15	65.2	17	68	32	66.7
	rs16975491	110833564	c.2193+75G>A	-	7	70	7	63.6	1	50	15	65.2	17	68	32	66.7
	rs10492497	110831866	c.2194–98A>G	-	3	30	4	36.4	1	50	8	34.8	6	24	14	29.2
	rs2131939	110831837	c.2194–69C>T	-	0	0	0	0.0	0	0	0	0.0	4	16	4	8.3
	rs503053	110831451	c.2345–68A>G	-	7	70	7	63.6	1	50	15	65.2	17	68	32	66.7
	-	110830612	c.2626–34T>C	-	0	0	0	0.0	0	0	0	0.0	1	4	1	2.1
	rs2305081	110830090	c.2716+99C>T	-	5	50	4	36.4	1	50	10	43.5	9	36	19	39.6
	rs1562173	110828922	c.2968+51C>T	-	7	70	7	63.6	1	50	15	65.2	17	68	32	66.7
	rs1975514	110828891	c.2969–31A>G	-	7	70	7	63.6	1	50	15	65.2	17	68	32	66.7
37	rs874204	110827580	c.3183G>A	Gly1061Gly	7	70	7	63.6	1	50	15	65.2	17	68	32	66.7
37	rs874203	110827574	c.3189A>T	Arg1063Arg	7	70	7	63.6	1	50	15	65.2	17	68	32	66.7
	-	110826231	c.3505+16C>T	-	0	0	0	0.0	0	0	0	0.0	1	4	1	2.1
	rs17517598	110825264	c.3506–147C>A	-	1	10	1	9.1	0	0	2	8.7	4	16	6	12.5
	rs2289799	110824974	c.3556+93G>C	-	7	70	7	63.6	1	50	15	65.2	17	68	32	66.7
	rs2275845	110823178	c.3557–99C>T	-	3	30	4	36.4	1	50	8	34.8	6	24	14	29.2
42	-	110822943	c.3693G>A	Thr1231Thr	0	0	2	18.2	1	50	3	13.0	7	28	10	20.8
	-	110822653	c.3742+231C>T	-	0	0	0	0.0	0	0	0	0.0	2	8	2	4.2
	rs589985	110819586	c.3877–9C>T	-	9	90	10	90.9	2	100	21	91.3	24	96	45	93.8
	rs1778817	110819460	c.3949+45C>T	-	9	90	10	90.9	2	100	21	91.3	24	96	45	93.8
	rs652572	110819457	c.3949+48T>C	-	9	90	10	90.9	2	100	21	91.3	24	96	45	93.8
	rs1213026	110819362	c.3949+143T>C	-	9	90	10	90.9	2	100	21	91.3	24	96	45	93.8
	-	110818760:110818763	c.3950–110_3950–113del4	-	0	0	0	0.0	0	0	0	0.0	1	4	1	2.1
45	rs3742207	110818598	c.4002A>C	Gln1334His	8	80	4	36.4	1	50	13	56.5	10	40	23	47.9
	rs1816884	110817171	c.4150+38C>G	-	4	40	6	54.5	2	100	12	52.2	14	56	26	54.2
	rs2298241	110816097	c.4151–189C>G	-	2	20	6	54.5	0	0	8	34.8	5	20	13	27.1
	rs2298240	110815673	c.4249+137G>C	-	0	0	0	0.0	0	0	0	0.0	2	8	2	4.2
	rs16975424	110814923	c.4250–134T>C	-	1	10	5	45.5	0	0	6	26.1	5	20	11	22.9
49	rs1133219	110813709	c.4470C>T	Ala1490Ala	3	30	5	45.5	2	100	10	43.5	10	40	20	41.7
	rs2275843	110813532	c.4640+7C>T	-	2	20	6	54.5	0	0	8	34.8	5	20	13	27.1
	-	110813531	c.4640+8G>A	-	4	40	2	18.2	0	0	6	26.1	0	0	6	12.5
	rs2275842	110813523	c.4640+16G>A	-	2	20	6	54.5	0	0	8	34.8	5	20	13	27.1
	rs617111	110807776	c.4641–32G>A	-	4	40	2	18.2	0	0	6	26.1	0	0	6	12.5
	rs681884	110805062	c.4756–209C>T	-	10	100	11	100.0	2	100	23	100.0	24	96	47	97.9
	-	110804970	c.4756–117G>C	-	1	10	1	9.1	0	0	2	8.7	0	0	2	4.2
51	rs650724	110804809	c.4800C>T	Ser1600Ser	2	20	4	36.4	0	0	6	26.1	11	44	17	35.4
	rs13260	110802123	c.*587C>A	-	2	20	4	36.4	0	0	6	26.1	11	44	17	35.4
	rs28362515	110801735	c.*975A>C	-	1	10	1	9.1	0	0	2	8.7	0	0	2	4.2
*COL4A2* (NM_001846.2)																
	rs7989823	110959643	c.-277A>C		7	70	9	81.8	0	0	16	69.6	25	100	41	85.4
	rs7990009	110959688	c.-232C>G		5	50	7	63.6	0	0	12	52.2	17	68	29	60.4
	rs7990017	110959705	c.-215C>T		7	70	9	81.8	0	0	16	69.6	23	92	39	81.3
	rs7991332	110959717	c.-203T>C		5	50	7	63.6	0	0	12	52.2	15	60	27	56.3
	rs35466678	110959787	c.-133A>G		3	30	3	27.3	0	0	6	26.1	14	56	20	41.7
	rs7327528	110960044	c.-44–163G>C		0	0	0	0.0	0	0	0	0.0	4	16	4	8.3
	rs76536922	110960164	c.-44–43C>T		0	0	0	0.0	0	0	0	0.0	4	16	4	8.3
	rs4773143	110960685	c.99+215T>C		7	70	7	63.6	1	50	15	65.2	21	84	36	75.0
	rs4773144	110960712	c.99+242A>G		7	70	7	63.6	1	50	15	65.2	21	84	36	75.0
	rs12876517	111009643	c.100–176G>A		6	60	9	81.8	2	100	17	73.9	19	76	36	75.0
	rs4771678	111076940	c.181–141T>C		8	80	10	90.9	2	100	20	87.0	20	80	40	83.3
Ex5	rs4238272	111077197	c.297G>A	Thr99Thr	10	100	11	100.0	2	100	23	100.0	22	88	45	93.8
	rs74967960	111077234	c.315+19T>C		0	0	0	0.0	0	0	0	0.0	1	4	1	2.1
	rs7334986	111080609	c.361–205G>A		7	70	4	36.4	1	50	12	52.2	8	32	20	41.7
	-	111080964	c.477+34C>T		3	30	4	36.4	1	50	8	34.8	0	0	8	16.7
	rs3929758	111082157	c.478–75C>A		9	90	10	90.9	2	100	21	91.3	22	88	43	89.6
Ex9	rs62621885	111082772	c.574G>T	Val192Phe	0	0	0	0.0	0	0	0	0.0	1	4	1	2.1
	rs60212072	111086650	c.685–98G>A		0	0	3	27.3	0	0	3	13.0	6	24	9	18.8
	rs41275108	111088456	c.727–160A>T		4	40	2	18.2	0	0	6	26.1	2	8	8	16.7
	rs7983487	111090854	c.862–111A>G		1	10	5	45.5	0	0	6	26.1	12	48	18	37.5
	rs7984937	111090909	c.862–56T>C		1	10	5	45.5	0	0	6	26.1	13	52	19	39.6
	rs7984100	111090924	c.862–41G>A		1	10	5	45.5	0	0	6	26.1	13	52	19	39.6
	rs7983979	111091024	c.912+9C>T		0	0	3	27.3	0	0	3	13.0	6	24	9	18.8
	rs4771680	111098017	c.958–159T>C		1	10	2	18.2	0	0	3	13.0	12	48	15	31.3
	rs7489705	111098110	c.958–66C>T		10	100	11	100.0	2	100	23	100.0	21	84	44	91.7
Ex17	rs4103	111098226	c.1008C>T	Pro336Pro	10	100	11	100.0	2	100	23	100.0	15	60	38	79.2
	rs59905747	111099045	c.1012–100C>G		5	50	5	45.5	1	50	11	47.8	5	20	16	33.3
	rs45612833	111099057	c.1012–88G>A		10	100	11	100.0	2	100	23	100.0	21	84	44	91.7
	rs7326449	111099122	c.1012–23G>A		10	100	11	100.0	2	100	23	100.0	21	84	44	91.7
	rs56676181	111101931	c.1079–95C>T		5	50	6	54.5	1	50	12	52.2	7	28	19	39.6
	rs75082326	111101952	c.1079–74A>G		5	50	6	54.5	1	50	12	52.2	7	28	19	39.6
Ex19	rs76425569	111102042	c.1095G>A	Pro365Pro	5	50	6	54.5	1	50	12	52.2	7	28	19	39.6
Ex19	rs74941798	111102126	c.1179C>T	Ile393Ile	5	50	6	54.5	1	50	12	52.2	7	28	19	39.6
	rs34734302	111102183	c.1189+47A>G		9	90	9	81.8	2	100	20	87.0	13	52	33	68.8
	-	111102853	c.1339+52C>G		0	0	0	0.0	0	0	0	0.0	1	4	1	2.1
	rs72657934	111102865	c.1339+64G>A		0	0	2	18.2	0	0	2	8.7	3	12	5	10.4
	rs9515218	111109859	c.1432+77A>G		9	90	10	90.9	2	100	21	91.3	16	64	37	77.1
	rs9555703	111109882	c.1432+100A>G		6	60	4	36.4	2	100	12	52.2	5	20	17	35.4
	rs9515219	111109960	c.1432+178T>C		9	90	10	90.9	2	100	21	91.3	16	64	37	77.1
	rs9521781	111111023	c.1433–95T>C		9	90	10	90.9	2	100	21	91.3	16	64	37	77.1
	rs9521782	111111043	c.1433–75G>A		9	90	10	90.9	2	100	21	91.3	15	60	36	75.0
Ex22	rs7990214	111111173	c.1488G>A	Pro496Pro	9	90	10	90.9	2	100	21	91.3	16	64	37	77.1
Ex22	rs7990383	111111235	c.1550G>A	Arg517Lys	9	90	10	90.9	2	100	21	91.3	16	64	37	77.1
	rs4773186	111111382	c.1596+101G>A		10	100	11	100.0	2	100	23	100.0	22	88	45	93.8
	rs41275110	111114554	c.1669+21G>A		4	40	6	54.5	0	0	10	43.5	6	24	16	33.3
	rs7992330	111114751	c.1776+20G>A		6	60	4	36.4	2	100	12	52.2	5	20	17	35.4
	rs3803237	111117668	c.1777–84G>A		4	40	6	54.5	0	0	10	43.5	6	24	16	33.3
	rs3803236	111117745	c.1777–7C>T		9	90	10	90.9	2	100	21	91.3	16	64	37	77.1
	rs3825490	111117984	c.1978+31C>T		4	40	4	36.4	1	50	9	39.1	8	32	17	35.4
	rs72657953	111118073	c.1978+120C>T		2	20	2	18.2	0	0	4	17.4	4	16	8	16.7
	rs1983931	111118102	c.1978+149G>A		9	90	10	90.9	2	100	21	91.3	16	64	37	77.1
	rs1983932	111118221	c.1979–129T>C		3	30	3	27.3	0	0	6	26.1	7	28	13	27.1
	rs41275112	111118450	c.2038+41C>T		0	0	0	0.0	0	0	0	0.0	1	4	1	2.1
	rs1927350	111118546	c.2038+137T>G		3	30	3	27.3	0	0	6	26.1	7	28	13	27.1
	rs3803232	111119296	c.2039–91A>G		9	90	10	90.9	2	100	21	91.3	16	64	37	77.1
	rs3803231	111119342	c.2039–45T>C		2	20	2	18.2	0	0	4	17.4	4	16	8	16.7
Ex27	rs3803230	111119396	c.2048G>C	Gly683Ala	2	20	2	18.2	0	0	4	17.4	4	16	8	16.7
	rs9559813	111121444	c.2096–120C>A		8	80	9	81.8	2	100	19	82.6	14	56	33	68.8
	rs9559814	111121483	c.2096–81A>G		9	90	10	90.9	2	100	21	91.3	16	64	37	77.1
Ex28	rs78829338	111121570	c.2102A>G	Lys701Arg	1	10	3	27.3	1	50	5	21.7	3	12	8	16.7
Ex28	rs9583500	111121620	c.2152C>T	Pro718Ser	4	40	4	36.4	0	0	8	34.8	5	20	13	27.1
	rs9515229	111121717	c.2203+46A>G		9	90	10	90.9	2	100	21	91.3	16	64	37	77.1
	rs9515230	111121847	c.2203+176T>C		2	20	2	18.2	0	0	4	17.4	4	16	8	16.7
	rs9588178	111125576	c.2425+79G>A		0	0	0	0.0	0	0	0	0.0	2	8	2	4.2
	rs11617206	111125606	c.2425+109A>G		7	70	7	63.6	1	50	15	65.2	22	88	37	77.1
	rs9588179	111125747	c.2425+250C>A		0	0	0	0.0	0	0	0	0.0	2	8	2	4.2
	rs9559818	111130226	c.2426–124G>A		8	80	7	63.6	1	50	16	69.6	21	84	37	77.1
	rs2281974	111130519	c.2587+8C>T		5	50	5	45.5	1	50	11	47.8	18	72	29	60.4
	rs9301457	111130599	c.2587+88G>C		10	100	11	100.0	2	100	23	100.0	25	100	48	100.0
	rs2281973	111130674	c.2587+163C>T		4	40	5	45.5	1	50	10	43.5	15	60	25	52.1
	rs72657977	111132413	c.2588–154C>T		4	40	3	27.3	0	0	7	30.4	3	12	10	20.8
	rs4773194	111132490	c.2588–77A>G		10	100	8	72.7	2	100	20	87.0	17	68	37	77.1
	rs9521803	111132556	c.2588–11C>T		8	80	6	54.5	2	100	16	69.6	8	32	24	50.0
	rs1475438	111132820	c.2758+83G>A		4	40	6	54.5	0	0	10	43.5	11	44	21	43.8
	rs58124222	111132947	c.2758+210G>A		0	0	3	27.3	0	0	3	13.0	8	32	11	22.9
	rs3803229	111134780	c.2759–83G>A		6	60	7	63.6	1	50	14	60.9	9	36	23	47.9
	rs3803228	111134858	c.2759–5T>C		0	0	3	27.3	0	0	3	13.0	8	32	11	22.9
	rs2296853	111137240	c.2903–12A>G		3	30	3	27.3	2	100	8	34.8	6	24	14	29.2
	rs2296852	111137465	c.3025+91G>A		2	20	2	18.2	0	0	4	17.4	10	40	14	29.2
	rs11839527	111137488	c.3025+114G>A		2	20	2	18.2	0	0	4	17.4	10	40	14	29.2
	rs41315048	111137975	c.3026–27G>T		1	10	3	27.3	0	0	4	17.4	2	8	6	12.5
	rs2296851	111138255	c.3207+72G>A		2	20	2	18.2	0	0	4	17.4	10	40	14	29.2
	rs35120918	111143541	c.3347–39G>A		0	0	1	9.1	1	50	2	8.7	0	0	2	4.2
	rs413756	111143755	c.3454+68T>C		8	80	11	100.0	1	50	20	87.0	22	88	42	87.5
	rs402661	111143851	c.3454+164G>C		3	30	5	45.5	0	0	8	34.8	10	40	18	37.5
	rs452020	111144102	c.3455–315T>C		10	100	11	100.0	2	100	23	100.0	25	100	48	100.0
	rs403839	111144321	c.3455–96G>A		2	20	2	18.2	0	0	4	17.4	7	28	11	22.9
	-	111144382	c.3455–35T>C		1	10	0	0.0	0	0	1	4.3	1	4	2	4.2
	rs2296849	111144412	c.3455–5C>G		1	10	3	27.3	0	0	4	17.4	2	8	6	12.5
	rs421177	111144565	c.3562+41C>T		1	10	3	27.3	0	0	4	17.4	2	8	6	12.5
	rs57003582	111145456:111145486	c.3563–100_3563–70del30		7	70	11	100.0	1	50	19	82.6	22	88	41	85.4	
	rs2274544	111145633	c.3634+4C>T		1	10	3	27.3	0	0	4	17.4	2	8	6	12.5
	rs2391833	111145676	c.3634+47G>C		8	80	10	90.9	1	50	19	82.6	20	80	39	81.3
	rs9559826	111145779	c.3634+150C>T		2	20	2	18.2	0	0	4	17.4	8	32	12	25.0
	-	111147637	c.3635–52A>G		3	30	4	36.4	1	50	8	34.8	0	0	8	16.7
	rs378601	111153934	c.3761–81G>A		10	100	11	100.0	2	100	23	100.0	25	100	48	100.0
	rs388222	111154159	c.3877+28C>T		8	80	8	72.7	2	100	18	78.3	21	84	39	81.3
	rs2281968	111154160	c.3877+29G>A		5	50	8	72.7	1	50	14	60.9	18	72	32	66.7
	rs4773198	111155711	c.4040–19C>T		5	50	6	54.5	1	50	12	52.2	8	32	20	41.7
Ex43	rs4773199	111155779	c.4089G>A	Ala1363Ala	5	50	4	36.4	1	50	10	43.5	8	32	18	37.5
	rs9301460	111156153	c.4139–41G>A		5	50	6	54.5	1	50	12	52.2	8	32	20	41.7
	rs414881	111156411	c.4285+71G>A		7	70	8	72.7	1	50	16	69.6	21	84	37	77.1
Ex45	rs4771683	111156499	c.4290T>C	Phe1430Phe	10	100	11	100.0	2	100	23	100.0	25	100	48	100.0
Ex46	rs445348	111158874	c.4515A>G	Pro1505Pro	10	100	11	100.0	2	100	23	100.0	25	100	48	100.0
	rs2479426	111164198	c.4882–83T>C		7	70	6	54.5	2	100	15	65.2	20	80	35	72.9
	rs422733	111164614	c.*76T>C		7	70	6	54.5	2	100	15	65.2	20	80	35	72.9
	rs3074455	111164639, 111164640	c.*101_*102del2		7	70	6	54.5	2	100	15	65.2	20	80	35	72.9
	rs10509	111164955	c.*417C>G		8	80	11	100.0	2	100	21	91.3	25	100	46	95.8
	rs1049906	111165079	c.*541C>T		4	40	8	72.7	1	50	13	56.5	18	72	31	64.6
	rs1049931	111165095	c.*557A>G		6	60	9	81.8	1	50	16	69.6	20	80	36	75.0
	rs1049977	111165188	c.*650T>C		6	60	9	81.8	1	50	16	69.6	19	76	35	72.9
	rs7711	111165201	c.*663T>C		6	60	9	81.8	1	50	16	69.6	19	76	35	72.9
	rs15457	111165265	c.*727G>C		4	40	8	72.7	1	50	13	56.5	17	68	30	62.5

dbSNP ref ID: identity numbers of observed sequence variants; chromosome position (NCBI build 37.1).

The sequencing of the genomic region containing the common promoter of *COL4A1* and *COL4A2* revealed no sequence changes.

### Statistical analysis and in silico predictions

**Figure 1:**
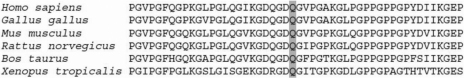
Multiple sequence alignment of the amino acid sequences of COL4A1 orthologs in different species. Conservation of glutamine (Q) at the 1334 position is shown in gray.

PolyPhen analyses of non-synonymous changes in COL4A1 and COL4A2 predicted that only the Gln1334His variant in COL4A1 was possibly damaging for protein function and structure (Table 3). The multiple sequence alignment of COL4A1 orthologs shows that the amino acid glutamine at position 1,334 is conserved throughout the analyzed species (Figure 1). Gln1334His substitution was observed more frequently in patients than in healthy individuals in family KTCN-014 (p=0.056). There was no difference in the c.4002A>C allele distribution between the analyzed affected individuals from the remaining KTCN families and the Ecuadorian control subjects (p=0.17).

**Table 3:** Prediction of effect of amino acid substitutions found in COL4A1 and COL4A2.

** **	** **	**PolyPhen**		**SIFT**		**PMUT**		**PANTHER**		**SNAP**	
**Gene**	**Sequence variant**	**PSIC score**	**Prediction**	**Score**	**Prediction**	**NN**	**Prediction**	**subPSEC**	**Pdeleterious**	**Expected Accuracy**	**Prediction**
COL4A1	Val7Leu	N/A	benign	1	tolerated	0.2367	neutral	-	-	92%	neutral
** **	Thr555Pro	N/A	benign	0.65	tolerated	0.0250	neutral	−0.52603	0.0777	94%	neutral
** **	Gln1334His	1.66	possibly damaging	0.12	tolerated	0.1039	neutral	−1.0433	0.12382	69%	neutral
COL4A2	Val192Phe	1.13	benign	64	tolerated	0.1921	neutral	-	-	78%	neutral
** **	Arg517Lys	0.1	benign	0.96	tolerated	0.0861	neutral	-	-	92%	neutral
** **	Gly683Ala	N/A	benign	0.96	tolerated	0.4841	neutral	-	-	85%	neutral
** **	Lys701Arg	N/A	benign	0.97	tolerated	0.0166	neutral	-	-	89%	neutral
** **	Pro718Ser	N/A	benign	0.98	tolerated	0.2039	neutral	-	-	89%	neutral

The PolyPhen tool predicts which missense substitution affects the structure and function of protein, and uses Position-Specific Independent Counts software to assign profile scores. The SIFT tool evaluates conserved positions, and calculates a score for the amino acid change at a particular position. A score of <0.05 is considered as pathogenic for the protein structure. The PMUT calculates the pathological significance of non-synonymous amino acid substitution using neural networks (NN). NN output >0.5 is considered to be deleterious. PANTHER generates the substitution Position-Specific Evolutionary Conservation score. The value −3 is cutoff point for functional significance and corresponds to a P_deleterious_ of 0.5. If the substitution occurs at a position not appearing in the multiple sequence alignment, a subPSEC score cannot be calculated and change is not likely to be pathogenic. The SNAP output shows prediction neutral or non-neutral, and the expected accuracy.

The SIFT, PMUT, PANTHER, and SNAP analyses defined all missense amino acid substitutions in COL4A1 and COL4A2 as neutral/tolerated and lacking any effect on protein function. All prediction results are summarized in Table 3.

### Haplotype reconstruction

**Figure 2:**
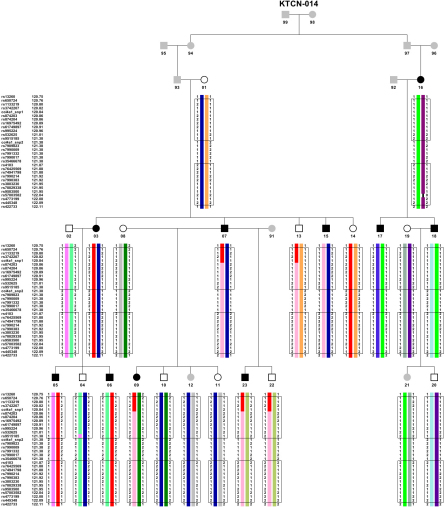
Pedigree of the family KTCN-014. Black-filled symbols: individuals with KTCN; open symbols: individuals without KTCN; gray-filled symbols: individuals with unknown KTCN status. Below each symbol the haplotypes are shown for the coding sequence in genes *COL4A1, COL4A2* and UTRs between them. In *COL4A1*, the coding regions are surrounded by the markers rs13260 and col4a2_snp, and by rs35466678 and rs422733 in *COL4A2,* which were marked by a black frame. Haplotype regions in different colors indicate patterns of inheritance in the two branches in the pedigree.

Haplotypes of sequence variants observed in family KTCN-014 are shown in Figure 2. The coding sequence variants in *COL4A1* are surrounded by markers rs13260 and col4a1_snp2. Exons of *COL4A2* are localized between rs35466678 and rs422733.

KTCN-014 consists of two family branches. Distinct haplotypes in the branches were identified (Figure 2). In the first one, initiated by parents KTCN-93 and KTCN-01, six subjects with KTCN had the same haplotype in the *COL4A1* region, extending from rs13260 to col4a1_snp1. Three unaffected individuals, KTCN-13, KTCN-14, and KTCN-22, share that part of the haplotype with their affected relatives. One of four variants in this region, rs3742207, causes a change in the protein sequence, replacing Gln in position 1334 with His (Gln1334His). That haplotype region, from rs13260 to col4a1_snp1, represents a short fragment of the haplotype which covers the whole *COL4A1* and *COL4A2* sequence in KTCN-03, KTCN-05, KTCN-06, and KTCN-14. In addition, individuals KTCN-07, KTCN-09, KTCN-13, KTCN-22, and KTCN-23 share the rs874203-rs422733 region (Figure 2 – pink bars). For markers rs13260-col4a1_snp1, a different haplotype was observed in the second family branch, initiated by parents KTCN-92 and KTCN-16. This haplotype covered the entire length of the analyzed region, and was identified in all affected individuals and KTCN-21, whose phenotype was unknown. Subject KTCN-17 had the same allele pattern for markers s13260-col4a1_snp1, as individuals from the first branch of the family. However, in this case, analysis indicated that these markers are inherited from KTCN-92, who is unrelated to KTCN-93 and KTCN-01.

## Discussion

To our knowledge, this is the first report describing complete sequence analysis of the coding regions and the exon-intron boundaries of *COL4A1* and *COL4A2* in families with KTCN. Previous studies have revealed a correlation between KTCN development and histopathological alterations in the structure of the corneal stroma and basement membrane, including a loss of collagen concentration [42] and rearrangement of collagen fibers [26]. Moreover, several types of collagen, including collagen type IV have been identified in the cornea [24], and *COL4A1* and *COL4A2* expression has been detected in the human cornea [29]. Finally, we had mapped a locus for KTCN to 13q32, in close proximity of which *COL4A1* and *COL4A2* are localized [21]. Given that information, we hypothesized that *COL4A1* and *COL4A2* genes are good candidates for causing KTCN in families with linkage to that locus.

Different studies have revealed several loci and a few candidate genes for familial KTCN. The first gene proposed as playing a significant role in KTCN pathogenesis was the *VSX1* (visual system homeobox 1, OMIM 605020) gene. It was suggested that a few disease-causing mutations were present in this gene [43,44], but recent studies have not confirmed these findings [21,45-47]. Next, heterozygous genomic 7-bp deletion in intron 2 of *SOD1* (superoxide dismutase 1; OMIM 147450) was identified in two families with KTCN [48,49]. In contrast, other studies have shown that mutations in this gene are not associated with KTCN pathogenesis [21,47]. Genetic analyses of *COL4A3*, *COL4A4*, *COL8A1*, and *COL8A2* genes have revealed no pathogenic mutations in patients with KTCN, indicating that other genetic factors cause the disease [50-52].

We identified several single base pair substitutions in the coding regions of *COL4A1* and *COL4A2*, including one novel heterozygous change, c.3693G>A in exon 42 of *COL4A1*. None of the detected alterations segregated fully with the affected phenotype in the analyzed members of the Ecuadorian KTCN families. Among the identified missense substitutions in *COL4A1*, one change, c.4002A>C (p. Gln1334His), was observed more frequently in KTCN patients than in healthy individuals in family KTCN-014. However, no significant statistical association of this change with familial disease could be proven (p=0.056), and no difference in the c.4002A>C allele distribution between the analyzed affected individuals from the remaining KTCN families and the Ecuadorian control subjects was discovered (p=0.17). To predict the impact of the substitutions on the structure and function of the protein, we used different tools. All identified missense substitutions in *COL4A1* and *COL4A2* were predicted by the SIFT, PMUT, PANTHER, and SNAP tools to have no effect, but PolyPhen defined the Gln1334His change in *COL4A1* as possibly damaging. Glutamine at this position is highly conserved in different species. Moreover, this change is present in the collagenous domain of the α1(IV) chain with Gly-X-Y repeats, which plays a role in the assembly into a triple-helical structure of the protein [22]. Replacement of the neutral residue (Gln) with the polar amino acid (His) at the Y position is likely to affect the protein structure. Nevertheless, further studies should be performed to determine the functional significance of this substitution.

To the best of our knowledge, no mutations in *COL4A1* were associated with corneal disease. The spectrum of *COL4A1*-related disorders included porencephaly (OMIM 175780) [53-55], Hereditary Angiopathy with Nephropathy, Aneurysm and Muscle Cramps (HANAC; OMIM 611773) [56], and brain small vessel disease with hemorrhage (OMIM 607595) [57]. Recent studies have also revealed an association between mutations in exon 29 of *COL4A1* and Axenfeld-Rieger anomaly with leukoencephalopathy and stroke [58]. In our study, none of the previously reported *COL4A1* mutations were identified. The absence of these changes in patients with KTCN suggests that they are specific to the above-mentioned disorders only, and are not associated with KTCN in the tested families. To date, no mutations responsible for *COL4A2-*related human diseases have been reported.

Besides changes identified in the coding regions of *COL4A1* and *COL4A2,* our study revealed numerous alterations in introns and UTRs of both genes, including single base pair substitutions, deletions, and insertions. Fourteen of these were novel and their clinical significance is not known. Each of the changes was observed in affected and healthy individuals in the tested families. Because important functional elements are located in non-coding regions of genes [59] and intronic alterations can result in a deleterious effect on pre-mRNA splicing [60], identification of these sequence variants could be non-accidental. Further research is needed to delineate the role of these sequence variants.

Recent studies have shown that a mouse with a mutation in a splice acceptor site of *Col4a1* has ocular dysgenesis. The mutation results in a lack of exon 40 from mice’s transcripts and leads to the accumulation of mis-folded protein in the lens epithelial cells. *Col4a1^∆ex40^* mice show optic nerve hypoplasia and anterior segment dysgenesis (ASD) including pigment dispersion, cataracts, and corneal opacifications [61]. Splice acceptor sites are highly conserved regions in different species [56]. We detected no alterations in the splice acceptor site in intron 39 of human *COL4A1*.

Extended genetic studies executed in families with KTCN have shown a high level of genetic heterogeneity [62]. The presence of many putative loci supports the hypothesis that KTCN is an oligogeneic disease in which accumulation of sequence variants at several loci cause a specific KTCN haplotype and may trigger the phenotypic effect. The absence of mutations in *COL4A1* and *COL4A2* genes indicates that other genes are involved in KTCN pathogenesis in Ecuadorian families.
